# Diagnosis and prevention of the vasodepressor type of neurally mediated syncope in Japanese patients

**DOI:** 10.1371/journal.pone.0251450

**Published:** 2021-06-25

**Authors:** Misaki Hasegawa, Tomoyoshi Komiyama, Kengo Ayabe, Susumu Sakama, Tetsuri Sakai, Kyong Hee Lee, Masahiro Morise, Atsuhiko Yagishita, Mari Amino, Ayumi Sasaki, Eiichiro Nagata, Hiroyuki Kobayashi, Koichiro Yoshioka, Yuji Ikari

**Affiliations:** 1 Department of Cardiology, Tokai University School of Medicine, Isehara, Kanagawa, Japan; 2 Department of Clinical Pharmacology, Tokai University School of Medicine, Isehara, Kanagawa, Japan; 3 Support Center for Medical Research and Education, Tokai University, Isehara, Kanagawa, Japan; 4 Department of Neurology, Tokai University School of Medicine, Isehara, Kanagawa, Japan; Medizinische Universitat Graz, AUSTRIA

## Abstract

We investigated circulatory dynamics in patients with vasodepressor type neurally mediated syncope (VT-NMS) by performing high-resolution Holter electrocardiography and a correlation analysis of changes in adenylate cyclase activity, blood pressure, and pulse during the head-up tilt test. Holter electrocardiography was performed for 30 patients. Adenylate cyclase activity was evaluated in lymphocytes from blood samples taken at rest and during the head-up tilt test. There was no change in autonomic nerve fluctuation during electrocardiography in VT-NMS patients, but our results showed a significant difference in blood pressure and adenylate cyclase activity between VT-NMS patients and healthy volunteers; the systolic blood pressure of VT-NMS patients decreased after 5 min, while at 10 min, the adenylate cyclase activity was the highest (0.53%) and the systolic blood pressure was the lowest (111.8 mm Hg). Pulse rates increased after 10 min. VT-NMS patients showed higher blood pressure, pulse rate, and adenylate cyclase activity during the tilt test than did healthy volunteers. In patients with syncope, standing for longer than 10 minutes may increase the risk of VT-NMS. From our results, we consider it likely that high systolic blood pressure and adenylate cyclase activity at rest cause fainting in VT-NMS patients. Our findings may be helpful for identifying individuals with a high risk of developing NMS in the healthy population.

## Introduction

Syncope is defined as “a loss of posture and a natural and complete recovery of consciousness as a result of transient loss of consciousness” [1, 2]. There are various pathological conditions that cause syncope, but the most common pathophysiology is transient hypoperfusion of the whole brain, and the causative diseases are diverse [1, 3, 4]. Although the frequency of unexplained causes varies across studies, neurally mediated syncope (NMS) is the most common [5–10], and has been recorded in 17%-37% of Japanese people [2, 11, 12]. Fainting from NMS occurs frequently and interferes with daily life [13, 14]; however, the cause is unknown due to recovery at the time of examination, and because the underlying mechanisms of onset and treatment are not clear [5, 15–18]. In addition, NMS itself is not a fatal disease, but because of the sudden onset, head injuries due to falls and traffic accidents are common [15, 19–23]. This means that not only the patient but also the people around them may be involved [15, 19, 20].

Previously, we have investigated the onset and causes of NMS by measuring adenylate cyclase (AC) activity at rest and during the head-up tilt (HUT) test [24, 25]. AC mediates the effects of the Gi protein α-subunit on blood vessel contraction and relaxation through interaction with the α1 or β2 adrenergic receptors. These previous studies also focused on the glutamic acid repeat polymorphism site at Glu 301–303 in the α2B-adrenergic receptor (*ADRA2B*) gene. This polymorphism has three genotypes: Glu12/12, Glu12/9, and Glu9/9. We found that Gi α-subunit binding was stronger in the Glu9 genotype than in Glu12, and this predicted changes in AC activity [25]. It is suggested that AC through its synthesis of cyclic adenosine monophosphate (cAMP) profoundly affects the contraction of vascular smooth muscle and is, thus, involved in syncope [24, 26–29]. Therefore, AC activity may be a useful parameter for assessing the risk of NMS with syncope in healthy people. Specifically, changes in AC activity at rest and during the HUT test in patients with the vasodepressor type (VT) of NMS were found to be significantly higher than those in healthy volunteers. These data suggest the possibility of AC activity being used for NMS diagnosis [24]. In addition, changes in the concentrations of norepinephrine and epinephrine during postural changes in the HUT test have been reported [30, 31]. Some studies have also reported that the catecholamine concentration was higher at rest than before fainting [30–33]. cAMP also reaches a high level before the patient faints [34–37].

A tilt test for syncope in high-risk individuals who have no single syncope or organic heart disease, or who have other types of syncope, is classified as Class I according to the Japanese Circulation Society (JCS) guidelines (http://j-circ.or.jp/english/). However, few facilities can perform tilt inspection, and hospitalization on the day before the test is required. In addition, this test places a heavy burden on the patient. Therefore, if it is possible to make a diagnosis by another less invasive test, diagnosis at more facilities may become possible.

Heart rate variability (HRV) is a convenient, non-invasive method that is useful for assessing the regulation of the autonomic nervous system of the heart [38–40]. HRV can also be evaluated using 24-hour Holter electrocardiography (ECG) and a short-term ECG. Our research group has also used 24-hour Holter ECG for arrhythmia diagnosis [41–43]. Since syncope involves the autonomic nerves [5], HRV results evaluated by time-domain and frequency-domain can be used as an index for diagnosing NMS. Studies using HRV indicate that patients with NMS have increased autonomic nervous activity in their daily lives. However, Sneddon et al. reported that there is no difference between NMS patients and healthy volunteers [44]. In addition, Lazzeri et al. showed low SDANN (standard deviation of the 5-min average NN intervals) levels of HRV for each syncope event [45]. These reports suggest syncytial involvement in syncope. High-resolution Holter ECG enables the evaluation of autonomic nerves by analyzing HRV.

This study included a large sample of VT-NMS patients and investigated the changes in circulatory dynamics in these patients by performing high-resolution Holter ECG and a correlation analysis of changes in AC activity, blood pressure (BP), and pulse rate during the HUT test. By clarifying the changes in pathological phenomena occurring during stress in VT-NMS patients, we examined the effectiveness of AC activity as a predictor of NMS recurrence and prognosis.

## Materials and methods

### Research patients

**Fig 1:**
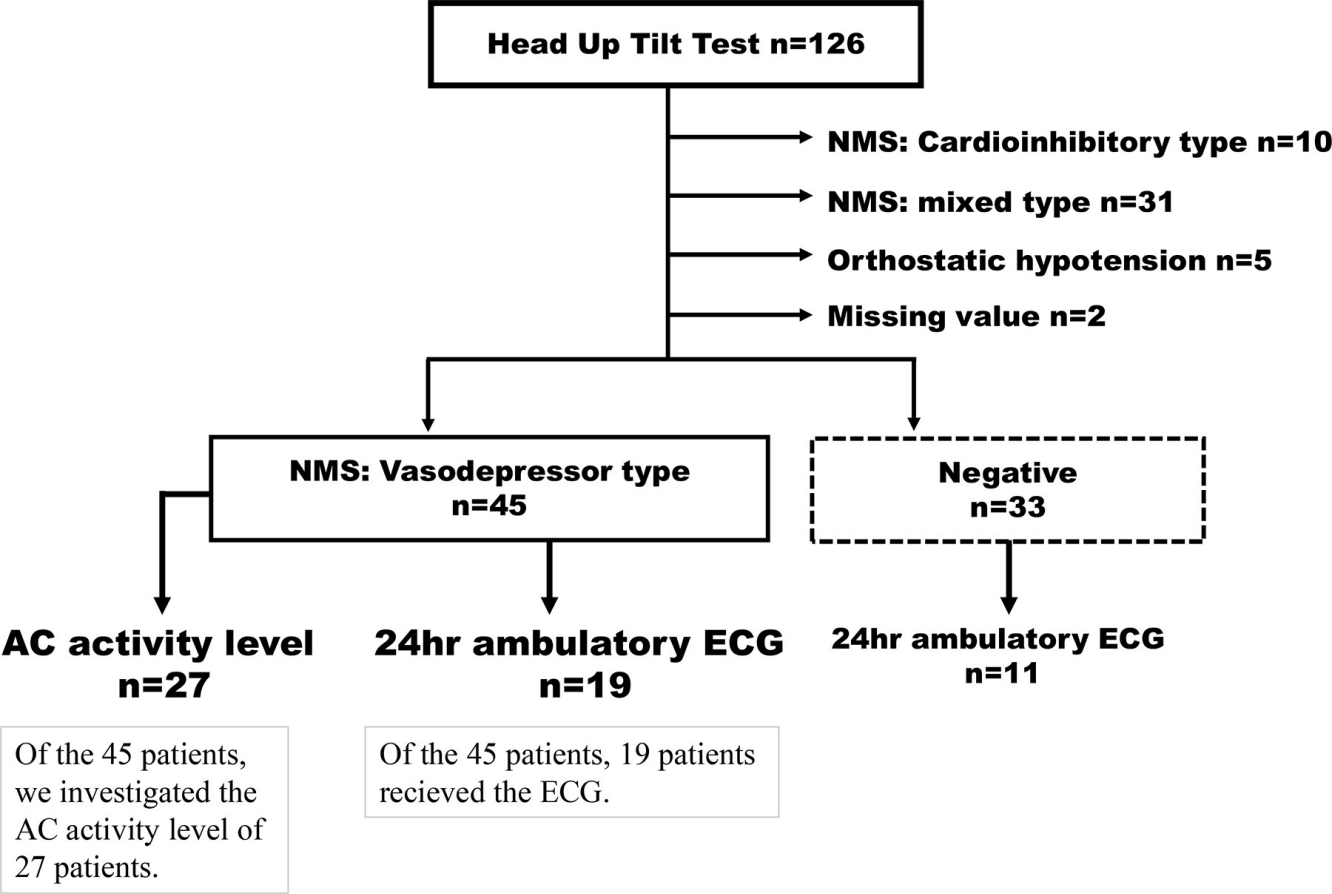
Schematic diagram of the survey target of our study. NMS, neurally mediated syncope; AC, adenylate cyclase; ECG, electrocardiogram.

Tilt tests were performed on 126 Japanese patients at our hospital between January 2016 and May 2020 (Table 1, S1 Table, and Fig 1). All these patients were suspected of having NMS with at least one loss of consciousness. All patients were examined and treated according to the guidelines of the JCS (http://j-circ.or.jp/english/). The Japanese guidelines follow the 2009 European Society of Cardiology guidelines [46].

**Table 1:** Background characteristics of NMS patients.

	All (n = 124)	VT-NMS (n = 45)	Negative (n = 33)
**Age (years), mean**	49.3±21.6	51.4±25.3	54.5±19.0
**Male, n (%)**	83 (66.9)	30 (66.7)	24 (72.7)
**Female, n (%)**	41 (33.1)	15 (33.3)	9 (27.3)
**EF (%), mean**	67.8±8.8	69.2±8.9	65.3±10.4
**(Base) Systolic BP (mmHg)**	119.6±17.8	118.5±14.1	124.3±21.7
**(Base) Diastolic BP (mmHg)**	73.7±12.6	74.4±10.2	76.1±15.6
**(Base) Pulse (bpm)**	66.4±14.0	66.3±14.4	66.5±12.5

NMS, neurally mediated syncope; VT, vasodepressor type; EF, ejection fraction; bpm, beats per min; BP, blood pressure.

Two of the 126 patients had missing data and were excluded from the target group. A retrospective comparative study was conducted on patients who underwent a tilt test. This resulted in 45 VT diagnoses and 33 negative tests.

A high-resolution Holter ECG (Digital Walk FM-180S, Fukuda Denshi, Tokyo, Japan; or Spider View, Ela Medical, Paris, France) was performed in 19 VT-NMS patients and 11 negative patients (Fig 1). A tilt test was then performed, and a retrospective comparative study was conducted on these 30 patients diagnosed with blood vessel inhibition type and 29 negative patients (Table 2).

**Table 2:** Background characteristics of VT-NMS patients and healthy volunteers for the tilt test.

	VT-NMS (n = 27)	Healthy (n = 15)	*p*-value
**Age**	43.8±21.3	37.2±7.8	NS (p = 0.256)
**Male (%)**	13 (48.2)	5 (33.3)	―
**Female (%)**	14 (51.8)	10 (66.7)	―
**(Rest time) Systolic BP (mm Hg)**	116.4±14.1	107.9±8.0	p = 0.0362
**(Rest time) Diastolic BP (mm Hg)**	74.0±12.0	66.2±8.8	p = 0.0322
**(Rest time) Pulse (bpm)**	66.7±14.1	65.7±8.9	NS (p = 0.818)

NMS, neurally mediated syncope; VT, vasodepressor type; BP, blood pressure; bpm, beats per min; NS, non-significant.

Diagnosis was also based on the guidelines of the JCS. The AC activity study compared VT-NMS patients and healthy volunteers. Patients with VT-NMS were hospitalized on the day before the test. After recording the patients’ medical history and medications and explaining the purpose and content of the test, we performed the HUT test. All patients and guardians of minors provided written and verbal consent for participation in the study prior to the test. Participating patients received verbal and written orientation to the study for over a period of one to two hours. All written consent was obtained in the form of signatures.

Patients diagnosed with VT-NMS by the HUT test were included in the study. We recruited healthy volunteers by using posters. Healthy volunteers provided informed consent after receiving the same information as the VT-NMS patients before the test. None of the patients had brain or heart disease.

This study was conducted under the approval of the Ethics Review Committee and the Medical Ethics Committee of Tokai University School of Medicine, Clinical Research Review Board (No. 14R-53) [24] and was in accordance with the Declaration of Helsinki. Only VT-NMS patients were used because a previous study provided further analysis of AC activity [24].

### Holter ECG

The Holter ECG was performed on 30 patients at the discretion of the outpatient attending physician (S2 Table). The test equipment, Digital Walk FM-180S (Fukuda Denshi, Tokyo, Japan) and Spider View (Ela Medical, Paris, France), was attached in the outpatient department, removed the next day, and analyzed.

### HUT test

The HUT test was performed in accordance with the guidelines. The patients were hospitalized on the day before the test and refrained from eating for 12 hours. The test was carried out between 9:00 and 11:00 the next morning [24].

**Fig 2:**
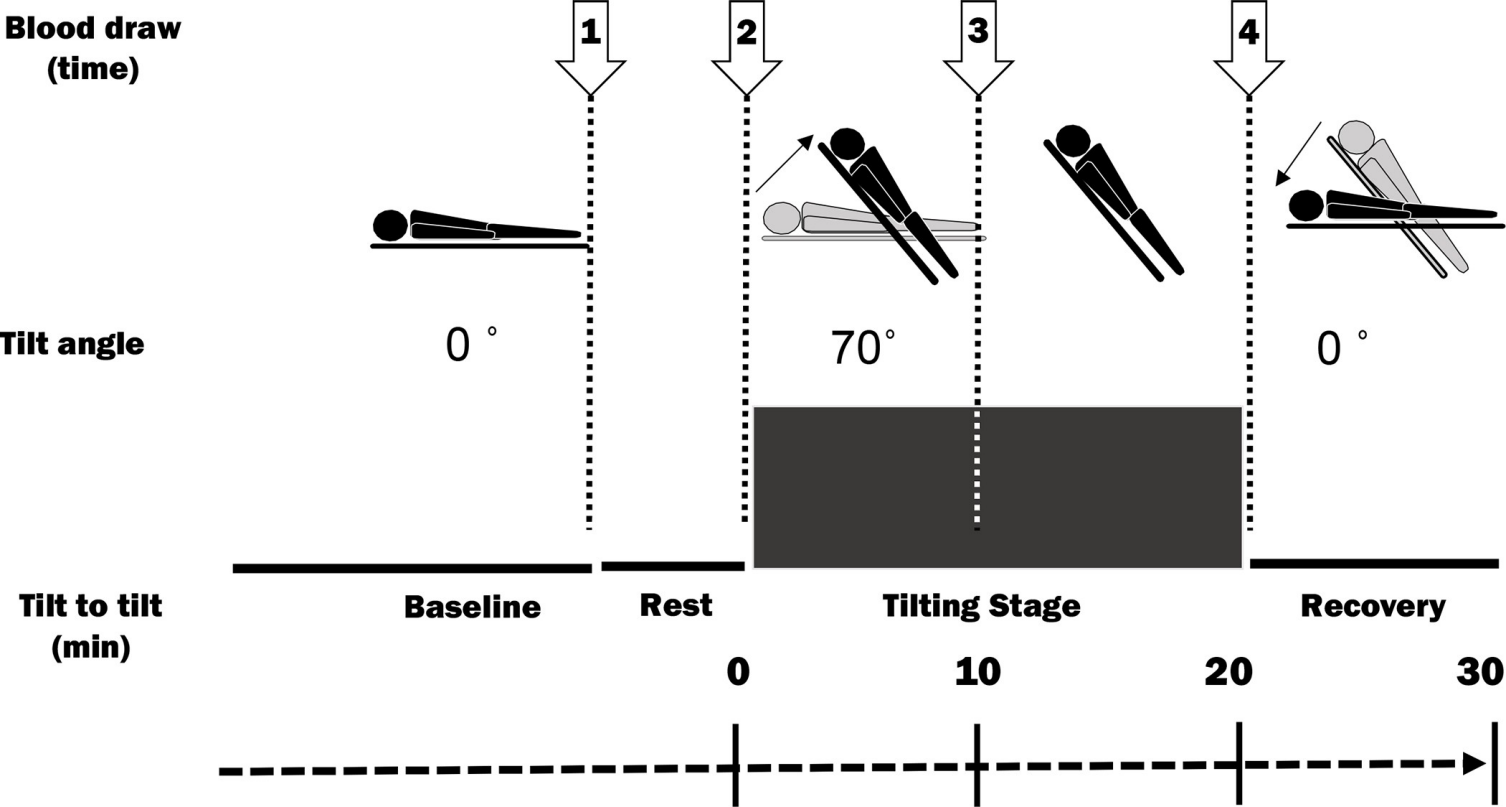
Picture of the head-up tilt table and four points of blood draw.

After resting for approximately 15–20 min in the supine position, the tilt table was elevated to 70°, and BP, pulse, and electrocardiograms were measured using Task Force Monitor TFM-3040 (Nihon Kohden, Tokyo, Japan) every min thereafter (Fig 2). The cuff was placed approximately 2 cm above the elbow. Patients who did not experience syncope were loaded with 0.3 mg of nitroglycerin (Toa Eiyo, Tokyo, Japan) 20 min after standing (Fig 2). The same procedure was performed on healthy volunteers.

### Comparison of AC activity in healthy volunteers and VT-NMS patients

Fifteen healthy volunteers (mean age, 37.2±7.8 years) and 27 VT-NMS patients (mean age, 43.8±21.3 years) were investigated. Patients were asked to refrain from caffeine and alcohol from the day before the test. The analysis data for VT-NMS patients did not include data after syncope.

### Blood sampling for measuring AC activity

We isolated the lymphocyte layer from the blood by centrifugation using Vacutainer Blood Collection Tubes (BD, New Jersey, NY, USA). Next, the lymphocytes were washed with induction buffer (RPMI 1640, Thermo Fisher Scientific, Inc., Waltham, MA, USA) in a medium to separate the platelets and isolate the lymphocytes. Lymphocytes were combined with the test reagent (adrenaline 10 μM for 10 000 cells) for 30 min at 25°C. cAMP was measured using the Promega cAMP-Glo Assay protocol (GloMax®-Multi Detection System, Wisconsin, USA) [47] and the values were confirmed using a standard curve [24]. AC activities were measured in the presence of induction buffer and /or test reagents (basal) and 100 μM forskolin (FK). In our study, FK concentrations reached a plateau at 100 μM FK. The results of the AD activity levels are expressed as percentages of FK-stimulated activity [24, 25, 48–50]:

The AC activity level was calculated as:

AC activity level = amount of cAMP produced of AD / amount of cAMP produced at 100 μM FK [24].

### Statistical analysis

JMP® 14 (SAS Institute Inc., Cary, NC, USA) was used for statistical analysis of AC activity, BP, and pulse rates. The analysis used a t-test to confirm a significant difference; p<0.05 was considered significant. The average variance was expressed as the mean ± standard deviation. In addition, for the analysis of the amount of AC activity, the ratio of AD 1 μM was determined with 100 μM FK as 100% [24].

## Results

### Patient background

The average age of the 124 patients was 49.3±21.6 years. The left ventricular ejection fraction measured by transthoracic echocardiography was 67.8%±8.8% (Table 1 and S1 Table). The average age of the patients with VT-NMS was 51.4±23.5 (men, 54.7±23.2; women, 44.9±23.6) years and of the negative patients was 54.5±19.0 (men, 57.4±19.3; women, 46.7±16.8) years. Both young and old individuals are thought to be susceptible to VT-NMS; however, there was no age difference in the cases suspected at our hospital. There were also no age-related differences in the ejection fraction between VT-NMS and negative patients (S3 Table). In addition, VT-NMS patients were not biased by age or sex. Moreover, there was no statistical difference, as VT-NMS tended to occur more frequently in older patients.

### Evaluation of high-resolution Holter ECG in VT-NMS and negative patients

Nineteen VT-NMS patients and 11 patients with negative HUT test results underwent a high-resolution ambulatory Holter ECG (S2 Table). The left ventricular ejection fraction measured by transthoracic echocardiography was 69.9%±8.8% for VT-NMS patients and 65.6%±11.0% for patients with negative tests. The number of premature ventricular contractions (PVCs) was significantly higher in the negative HUT group than in the VT-NMS group (611.7±1061.9 vs. 6.6±10.7; p = 0.018).

### Comparison of the AC activity level at rest and the timing of the HUT test between VT-NMS patients and healthy volunteers

**Fig 3:**
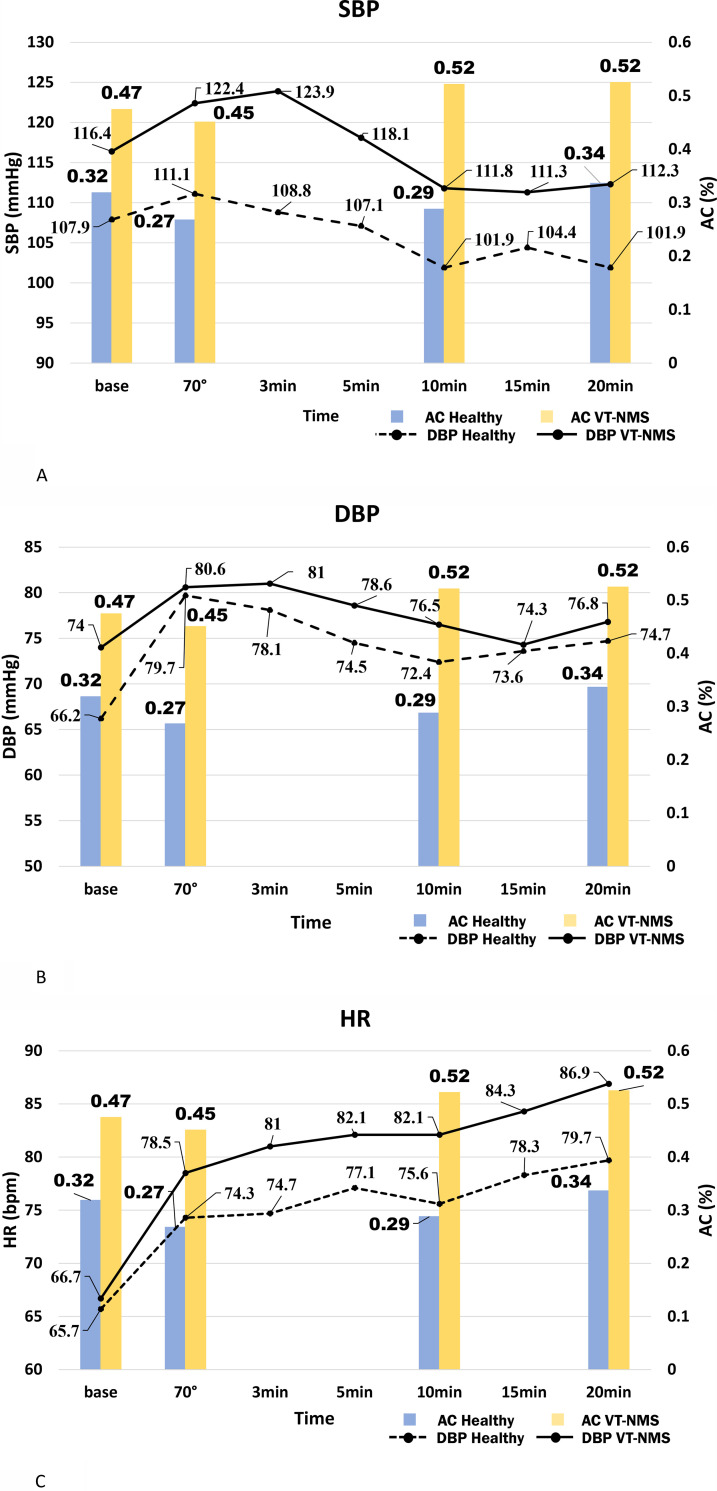
Changes in BP, HR, and AC activity in VT-NMS patients and healthy volunteers during tilt tests. (a) SBP, (b) DBP, (c) HR. BP, blood pressure; SBP, systolic blood pressure; DBP, diastolic blood pressure; HR, heart rate; AC, adenylate cyclase; VT, vasodepressor type; NMS, neurally mediated syncope.

The systolic pressure at rest (seven points) was significantly higher in the VT-NMS group than in the healthy volunteers (116.4±14.1 mm Hg vs 107.9±8.0 mm Hg; p = 0.0362) (Tables 2–4; S4 Table; and Fig 3). During the HUT test, BP, and heart rate (HR) increased in both the VT-NMS patients and the patients who received negative HUT test results (S5 Table and Fig 3). BP and HR of both groups were also higher in comparison with the healthy volunteer group. In particular, SBP was significantly higher (p = 0.036) in patients with VT-NMS (Fig 3A). The diastolic blood pressure (DBP) was also significantly higher (p = 0.032) in patients with VT-NMS. Moreover, the level of AC activity was consistently higher in the VT-NMS group at any point during the entire course of the HUT test (at rest, standing, 10 min after standing, and 20 min after standing) (Table 3).

**Table 3:** AC activities of VT-NMS patients and healthy volunteers.

	Base	70°	10 min	20 min
**15 healthy volunteers**	0.32±0.14	0.27±0.14	0.29±0.10	0.34±0.12
**27 VT patients**	0.47±0.18	0.45±0.19	0.52±0.21	0.52±0.21
***P* value**	0.0051	0.0020	0.0002	0.0031

AC, adenylate cyclase; VT, vasodepressor type; NMS, neurally mediated syncope.

**Table 4:** Data for 27 VT-NMS patients and 15 healthy volunteers.

a. SBP	Base	70°	3 min	5 min	10 min	15 min	20 min
**VT patients**	116.4±14.1	122.4±14.0	123.9±18.4	118.1 ±16.6	111.8±19.8	111.3±21.4	112.3±16.9
**Rate of change**		(6.0)	(7.5)	(1.7)	(-4.6)	(-5.1)	(-4.1)
**Healthy volunteers**	107.9±8.0	111.1±15.5	108.8±9.1	107.1±12.6	101.9±10.0	104.4±10.7	101.9±9.1
**Rate of change**		(3.2)	(0.9)	(-0.8)	(-6.0)	(-3.5)	(-6.0)
***p* value**	0.036	0.020	0.019	0.032	0.090	0.264	0.037
**b. DBP**	**Base**	**70°**	**3 min**	**5 min**	**10 min**	**15 min**	**20 min**
**VT patients**	74.0±12.0	80.6±11.9	81.0±11.6	78.6±11.8	76.5±13.5	74.3±14.4	76.8±15.1
**Rate of change**		(6.6)	(7.0)	(4.6)	(2.5)	(0.3)	(2.8)
**Healthy volunteers**	66.2±8.8	79.7±8.5	78.1±7.8	74.5±8.1	72.4±10.6	73.6±10.7	74.7±8.7
**Rate of change**		(13.5)	(11.9)	(8.3)	(6.2)	(7.4)	(8.5)
***p* value**	0.032	0.815	0.472	0.248	0.335	0.865	0.640
**c. HR**	**Base**	**70°**	**3 min**	**5 min**	**10 min**	**15 min**	**20 min**
**VT patients**	66.7±14.1	78.5±16.8	81.0±15.8	82.1±17.0	82.1±18.1	84.3±19.6	86.9±21.6
**Rate of change**		(11.8)	(14.3)	(15.4)	(15.4)	(17.6)	(20.2)
**Healthy volunteers**	65.7±8.9	74.3±7.6	74.7±6.8	77.1±7.4	75.6±7.4	78.3±8.5	79.7±8.0
**Rate of change**		(13.5)	(11.9)	(8.3)	(6.2)	(7.4)	(8.5)
***p* value**	0.818	0.360	0.233	0.282	0.212	0.282	0.232

NMS, neurally mediated syncope; VT, vasodepressor type; SBP, systolic blood pressure; DBP, diastolic blood pressure; HR, heart rate.

Our results showed that AC activity levels of the VT-NMS patients were significantly higher (p<0.05) than those of the healthy volunteers in both the rest position and at the four points (baseline, 70°, after 10 min, after 20 min) of the HUT test position (Tables 3 and 4 and Fig 3). Additionally, the amount of AC activity after 10 min had the most significant difference (p = 0.0002) between the healthy volunteers and VT-NMS patients. The AC activity immediately after stress loading decreased in both the healthy volunteers and VT-NMS patients; however, the BP increased.

In the healthy volunteers, we were not able to confirm significant differences in BP and AC activity. However, in the VT-NMS patients, the highest systolic BP (SBP) (123.9±18.4 mm Hg) was reached 3 min after stress loading, decreased after 5 min, and decreased further to 111.8 ±19.8 mm Hg after 10 min (rate of change, -12.1 mmHg) (Table 4 and Fig 3A). The BP did not change from 10 to 20 min; however, AC activity was higher than that at rest. During diastole, the BP of the healthy volunteers and VT-NMS patients increased immediately after stress loading but no significant changes were observed thereafter. In addition, the pulse rate increased significantly in both the healthy volunteers and VT-NMS patients immediately after stress; however, the healthy volunteers did not elicit significant differences 20 min after stress. However, in the VT-NMS patients, it increased further after 10 min and reached 86.9±21.6 bpm after 20 min. In the tilt test, AC decreased after 10 min, BP decreased, and pulse rate increased.

## Discussion

In our NMS examination, we focused on the most common VT-NMS and evaluated autonomic nerves during non-seizures together with the high-resolution Holter ECG and the HUT test. Among the 124 patients, there was no predominant age for NMS; as it affects young and old individuals, there was no age difference in the cases suspected at our hospital. In addition, VT-NMS patients were not biased by sex.

Moreover, in the analysis of the high-resolution Holter ECG, we did not observe a change in autonomic nerve fluctuation in VT-NMS patients. In our hospital, a 24-hour Holter ECG is performed to rule out fainting due to arrhythmia. This has been proposed as a non-invasive risk assessment for sudden death [42, 43]. In addition, it is possible that negative patients may have a hidden disease that may cause sudden death, and further scrutiny, such as an implantable loop recorder, is considered necessary [51–55].

Moreover, the number of PVCs recorded for the 24-hour Holter ECG was significantly higher (p = 0.018) in negative patients, and three of the negative patients had high levels (S2 Table). None of the three patients took beta blockers, and the results only occurred in approximately 1%-3% of the total HR measurements. In general, PVC itself has a good prognosis; however, there are case reports that it may trigger serious arrhythmias [56]. In our study, one of the three patients died suddenly of unknown cause. The relationship between syncope and arrhythmia has rarely been diagnosed by Holter [57]. Therefore, in the future, long-term monitoring needs to be considered to analyze the cause of syncope in negative patients.

In the HUT test, the SBP decreased from the HUT standing position (70°) after 10 min, and the hemodynamics were affected, even before syncope. In VT-NMS patients, it is considered that the blood vessels dilate and syncope occurs because of an increase in AC activity. From our results, it can be said that VT-NMS patients are prone to fainting due to high SBP and AC activity at rest. Patients with syncope who have high AC activity and high SBP at rest are more likely to be NMS-positive. These results support our previous findings and may, therefore, be one of the indicators when performing diagnosis and treatment in facilities where the tilt test cannot be performed. Since VT-NMS patients have high resting SBP and AC activity, these patients should be given lifestyle guidance that does not aggravate AC activity (Fig 3A). Our results showed that AC activity levels of VT -NMS patients were significantly higher than those of the healthy volunteers in both the rest position and at the four points of the HUT position (Table 3). In particular, the amount of AC activity after 10 min was the most significant difference (p = 0.0002) between healthy volunteers and VT-NMS patients. The AC activity immediately after stress loading decreased in both healthy volunteers and VT-NMS patients; however, the SBP increased. In addition, the DBP of the VT-NMS patients increased further from 10 min (Fig 3A and 3B).

In patients with syncope, standing for more than 10 min may increase the risk of VT-NMS. AC synthesizes cAMP from ATP [58–60]. At that time, owing to the action of the α2B-adrenergic receptor, the Gi protein α-subunit suppresses AC activity and causes blood vessel contraction [48, 49, 61–63]. On the other hand, the β2 adrenergic receptor signals the blood vessels to relax by binding to the Gs protein [49, 64]. In addition, when cAMP activates protein kinase A, the calcium ion channel opens and calcium uptake is promoted, which affects the contractile force of smooth muscle [26–29, 65–67]. Therefore, an increase in AC activity results in a decrease in SBP. In VT-NMS patients, when the AC activity increased before NMS syncope, the SBP decreased. Healthy patients have stable AC levels and do not have large fluctuations in SBP. For VT-NMS patients, when the blood vessels dilate and syncope occurs, this is caused by an increase in AC activity (rate of change, +0.07%) (Fig 3A). From our results, we consider it likely that high SBP and AC activity at rest cause fainting in VT-NMS patients. In the future, we would like to increase our number of VT-NMS patients and investigate other NMS types, such as the mixed-type and cardioinhibitory type.

Patients with syncope who have high AC activity and high SBP at rest are more likely to be VT- NMS-positive (Fig 3A). It is thought that providing guidance to patients with suspected VT- NMS will help prevent syncope; such guidance includes advice to avoid dehydration and stressful situations, such as standing for more than 10 min, and hot and crowded environments. In addition, various studies have connected caffeine intake to increased AC activity [68–70], and it is necessary to instruct these patients to refrain from caffeine intake. Although there are no studies on the effectiveness of providing such lifestyle guidance, reduced caffeine intake has a significant impact on reducing the recurrence of syncope [1].

Our study had some limitations. While we confirmed that there are differences in the patterns of AC activity, BP, and HR in VT- and mixed-type NMS patients, especially those with VT-NMS, we plan to further analyze mixed-type patients. Since the number of cardioinhibitory type patients in this study was only 10, a further survey should be conducted with a larger cohort. After considering the three types of NMS, we decided to focus this study on the VT-NMS type because we were able to survey more VT-NMS patients, and these patients showed significantly higher AC activities and BP values.

## Conclusions

Our study showed a significant difference in SBP and AC activities between VT-NMS patients and healthy volunteers during the HUT test. In VT-NMS patients, when the AC activity increased before NMS syncope, the SBP decreased. Conversely, healthy volunteers have stable AC activity levels and do not experience large fluctuations in SBP. For VT-NMS patients, when blood vessels dilate and syncope occurs, the cause is an increase in AC activity. For that reason, in patients with syncope, standing for more than 10 min may increase the risk of VT-NMS. From our results, we consider it likely that high SBP and AC activity at rest cause fainting in VT-NMS patients. In addition, our results offer a means of identifying people in the healthy population that are at risk of developing NMS.

## Supporting information

S1 Table Characteristics of all patients. (DOCX)

S2 Table Raw data for Holter ECG of 30 VT -NMS patients. (DOCX)

S3 Table Analysis of EF differences by age group between VT-NMS-positive and VT-NMS-negative patients. (DOCX)

S4 Table Rate of change of SBP, DBP, and HR in VT-NMS patients and healthy volunteers. (DOCX)

S5 Table Raw data for adenylate cyclase activities in healthy volunteers and VT-NMS patients during the HUT test. (DOCX)

## References

[1] Brignole M, Moya A, de Lange FJ, Deharo JC, Elliott PM, Fanciulli A, et al. 2018 ESC Guidelines for the diagnosis and management of syncope. Eur Heart J. 2018;39(21):1883–1948. doi: 10.1093/eurheartj/ehy037 29562304

[2] Tanimoto K, Yukiiri K, Mizushige K, Takagi Y, Masugata H, Shinomiya K, et al. Usefulness of brain natriuretic peptide as a marker for separating cardiac and noncardiac causes of syncope. Am J Cardiol. 2004;93(2):228–230. doi: 10.1016/j.amjcard.2003.09.048 14715356

[3] Hainsworth R. Pathophysiology of syncope. Clin Auton Res. 2004;14(Suppl 1):18–24. doi: 10.1007/s10286-004-1004-2 15480926

[4] Saklani P, Krahn A, Klein G. Syncope. Circulation. 2013;127(12):1330–1339. doi: 10.1161/CIRCULATIONAHA.112.138396 23529534

[5] Mosqueda-Garcia R, Furlan R, Tank J, Fernandez-Violante R. The elusive pathophysiology of neurally mediated syncope. Circulation. 2000;102(23):2898–2906. doi: 10.1161/01.cir.102.23.2898 11104751

[6] Zaqqa M, Massumi A. Neurally mediated syncope. Tex Heart Inst J. 2000;27(3):268–272. 11093411 PMC101078

[7] Kapoor WN. Evaluation and outcome of patients with syncope. Medicine (Baltimore). 1990;69(3):160–175. doi: 10.1097/00005792-199005000-00004 2189056

[8] Linzer M, Yang EH, Estes MNA 3rd, Wang P, Vorperian VR, Kapoor WN. Clinical guideline: diagnosing syncope: part 1: value of history, physical examination, and electrocardiography. Ann Intern Med. 1997;126(12):989–996. doi: 10.7326/0003-4819-126-12-199706150-00012 9182479

[9] Liao D, Xu Y, Zou R, Wu L, Luo X, Li F, et al. The circadian rhythm of syncopal episodes in patients with neurally mediated syncope. Int J Cardiol. 2016;215:186–192. doi: 10.1016/j.ijcard.2016.04.086 27128529

[10] Chu W, Wang C, Lin P, Li F, Wu L, Xie Z. Transient aphasia: a rare complication of head-up tilt test. Neurol Sci. 2014;35(7):1127–1132. doi: 10.1007/s10072-014-1664-1 24514919

[11] Suzuki M, Hori S, Nakamura I, Soejima K, Aikawa N. Long-term survival of Japanese patients transported to an emergency department because of syncope. Ann Emerg Med. 2004;44(3):215–221. doi: 10.1016/j.annemergmed.2004.02.036 15332061

[12] Onuki T, Ito H, Ochi A, Chiba Y, Kawasaki S, Onishi Y, et al. Single center experience in Japanese patients with syncope. J Cardiol. 2015;66(5):395–402. doi: 10.1016/j.jjcc.2014.12.009 25736069

[13] Alboni P. The different clinical presentations of vasovagal syncope. Heart. 2015;101(9):674–678. doi: 10.1136/heartjnl-2014-307096 25792719

[14] Ungar A, Sgobino P, Russo V, Vitale E, Sutton R, Melissano D, et al. Diagnosis of neurally mediated syncope at initial evaluation and with tilt table testing compared with that revealed by prolonged ECG monitoring. An analysis from the Third International Study on Syncope of Uncertain Etiology (ISSUE-3). Heart. 2013;99(24):1825–1831. doi: 10.1136/heartjnl-2013-304399 24153416

[15] Martin K, Bates G, Whitehouse WP. Transient loss of consciousness and syncope in children and young people: what you need to know. Arch Dis Child Educ Pract Ed. 2010;95(3):66–72. doi: 10.1136/adc.2007.121103 20501529

[16] Mercader MA, Varghese PJ, Potolicchio SJ, Venkatraman GK, Lee SW. New insights into the mechanism of neurally mediated syncope. Heart. 2002;88(3):217–221. doi: 10.1136/heart.88.3.217 12181208 PMC1767328

[17] Silvani S, Padoan G, Guidi AR, Bianchedi G, Maresta A. Cerebral vasoconstriction in neurally mediated syncope: relationship with type of head-up tilt test response. Ital Heart J. 2003;4(11):768–775. 14699706

[18] Schondorf R, Stein R, Roberts R, Benoit J, Cupples W. Dynamic cerebral autoregulation is preserved in neurally mediated syncope. J Appl Physiol (1985). 2001;91(6):2493–2502. doi: 10.1152/jappl.2001.91.6.2493 11717210

[19] Jhanjee R, Van Dijk JG, Sakaguchi S, Benditt DG. Syncope in adults: terminology, classification, and diagnostic strategy. Pacing Clin Electrophysiol. 2006;29(10):1160–1169. doi: 10.1111/j.1540-8159.2006.00508.x 17038147

[20] Iglesias JF, Graf D, Forclaz A, Schlaepfer J, Fromer M, Pruvot E. Stepwise evaluation of unexplained syncope in a large ambulatory population. Pacing Clin Electrophysiol. 2009;32:S202–S206. doi: 10.1111/j.1540-8159.2008.02291.x 19250095

[21] Varga E, Wórum F, Szabó Z, Varga M, Lõrincz I. Motor vehicle accident with complete loss of consciousness due to vasovagal syncope. Forensic Sci Int. 2002;130(2–3):156–159. doi: 10.1016/s0379-0738(02)00377-8 12477637

[22] Hadjikoutis S, O’Callaghan P, Smith PE. The investigation of syncope. Seizure. 2004;13(8):537–548. doi: 10.1016/j.seizure.2003.12.011 15519913

[23] Parry SW, Reeve P, Lawson J, Shaw FE, Davison J, Norton M, et al. The Newcastle protocols 2008: an update on head-up tilt table testing and the management of vasovagal syncope and related disorders. Heart. 2009;95(5):416–420. doi: 10.1136/hrt.2007.136457 18701533

[24] Komiyama T, Nagata E, Hashida T, Sakama S, Ayabe K, Kamiguchi H, et al. Neurally mediated syncope diagnosis based on adenylate cyclase activity in Japanese patients. PloS one. 2019;14(4):e0214733. doi: 10.1371/journal.pone.0214733 30998713 PMC6472876

[25] Komiyama T, Hirokawa T, Sato K, Oka A, Kamiguchi H, Nagata E, et al. Relationship between human evolution and neurally mediated syncope disclosed by the polymorphic sites of the adrenergic receptor gene α2B-AR. PloS one. 2015;10(4):e0120788. doi: 10.1371/journal.pone.0120788 25860977 PMC4393242

[26] Lincoln TM, Cornwell TL, Taylor AE. cGMP-dependent protein kinase mediates the reduction of Ca2+ by cAMP in vascular smooth muscle cells. Am J Physiol. 1990;258(3 Pt 1):C399–C407. doi: 10.1152/ajpcell.1990.258.3.C399 2156436

[27] Ishikawa T, Hume JR, Keef KD. Regulation of Ca2+ channels by cAMP and cGMP in vascular smooth muscle cells. Circ Res. 1993;73(6):1128–1137. doi: 10.1161/01.res.73.6.1128 8222084

[28] Keef KD, Hume JR, Zhong J. Regulation of cardiac and smooth muscle Ca(2+) channels (Ca(V)1.2a,b) by protein kinases. Am J Physiol Cell Physiol. 2001;281(6):C1743–C1756. doi: 10.1152/ajpcell.2001.281.6.C1743 11698232

[29] Itoh T, Ikebe M, Kargacin GJ, Hartshorne DJ, Kemp BE, Fay FS. Effects of modulators of myosin light-chain kinase activity in single smooth muscle cells. Nature. 1989;338(6211):164–167. doi: 10.1038/338164a0 2493140

[30] Kikushima S, Kobayashi Y, Nakagawa H, Katagiri T. Triggering mechanism for neurally mediated syncope induced by head-up tilt test: role of catecholamines and response to propranolol. J Am Coll Cardiol. 1999;33(2):350–357. doi: 10.1016/s0735-1097(98)00567-1 9973014

[31] Sra JS, Murthy V, Natale A, Jazayeri MR, Dhala A, Deshpande S, et al. Circulatory and catecholamine changes during head-up tilt testing in neurocardiogenic (vasovagal) syncope. Am J Cardiol. 1994;73(1):33–37. doi: 10.1016/0002-9149(94)90723-4 8279374

[32] Fitzpartick A, Williams T, Ahmed R, Lightman S, Bloom SR, Sutton R. Echocardiographic and endocrine changes during vasovagal syncope induced by prolonged head-up tilt. European Journal of Cardiac Pacing and Electrophysiology. 1992;2:121–128.

[33] Goldstein DS, Spanarkel M, Pitterman A, Toltzis R, Gratz E, Epstein S, et al. Circulatory control mechanisms in vasodepressor syncope. Am Heart J. 1982;104(5):1071–1075. doi: 10.1016/0002-8703(82)90442-2 7136999

[34] Mitro P, Rybárová E, Žemberová E, Tkáč I. Enhanced plasma catecholamine and cAMP response during the head-up tilt test in patients with vasovagal syncope. Wien Klin Wochenschr. 2005;117(9–10):353–358. doi: 10.1007/s00508-005-0331-1 15989115

[35] Abe H, Kobayashi H, Nakashima Y, Izumi F, Kuroiwa A. Plasma catecholamines and cyclic AMP response during head-up tilt test in patients with neurocardiogenic (vasodepressor) syncope. Pacing Clin Electrophysiol. 1995;18(7):1419–1426. doi: 10.1111/j.1540-8159.1995.tb02604.x 7567595

[36] Baillie GS, Sood A, McPhee I, Gall I, Perry SJ, Lefkowitz RJ, et al. β-Arrestin-mediated PDE4 cAMP phosphodiesterase recruitment regulates β-adrenoceptor switching from Gs to Gi. Proc Natl Acad Sci U S A. 2003;100(3):940–945. doi: 10.1073/pnas.262787199 12552097 PMC298705

[37] Nguyen K, Kassimatis T, Lymperopoulos A. Impaired desensitization of a human polymorphic α2B-adrenergic receptor variant enhances its sympatho-inhibitory activity in chromaffin cells. Cell Commun Signal. 2011;9(1):5. doi: 10.1186/1478-811X-9-5 21299895 PMC3041786

[38] Heart rate variability: standards of measurement, physiological interpretation and clinical use. Task Force of the European Society of Cardiology and the North American Society of Pacing and Electrophysiology. Circulation. 1996;93(5):1043–65. 8598068

[39] Kochiadakis GE, Kanoupakis EM, Rombola AT, Igoumenidis NE, Chlouverakis GI, Vardas PE. Reproducibility of tilt table testing in patients with vasovagal syncope and its relation to variations in autonomic nervous system activity. Pacing Clin Electrophysiol. 1998;21(5):1069–1076. doi: 10.1111/j.1540-8159.1998.tb00152.x 9604238

[40] Hosaka H, Takase B, Katsushika S, Ohsuzu F, Kurita A. Altered fractal behavior and heart rate variability in daily life in neurally mediated syncope. Biomed Pharmacother. 2003;57(Suppl 1):77s–82s. doi: 10.1016/j.biopha.2003.08.009 14572680

[41] Yoshioka K, Amino M, Zareba W, Shima M, Matsuzaki A, Fujii T, et al. Identification of high-risk Brugada syndrome patients by combined analysis of late potential and T-wave amplitude variability on ambulatory electrocardiograms. Circ J. 2013;77(3):610–618. doi: 10.1253/circj.cj-12-0932 23439592

[42] Amino M, Yoshioka K, Morita S, Iizuka S, Otsuka H, Yamamoto R, et al. One-year follow-up and convalescence evaluated by nuclear medicine studies and 24-hour holter electrocardiogram in 11 patients with myocardial injury due to a blunt chest trauma. J Trauma. 2009;66(5):1308–1310. doi: 10.1097/TA.0b013e31817e0f46 19430231

[43] Amino M, Yoshioka K, Ichikawa T, Watanabe E, Kiyono K, Nakamura M, et al. The presence of late potentials after percutaneous coronary intervention for the treatment of acute coronary syndrome as a predictor for future significant cardiac events resulting in re-hospitalization. J Electrocardiol. 2019;53:71–78. doi: 10.1016/j.jelectrocard.2019.01.003 30703576

[44] Sneddon JF, Bashir Y, Murgatroyd FD, Ward DE, Camm AJ, Malik M. Do patients with neurally mediated syncope have augmented vagal tone? Am J Cardiol. 1993;72(17):1314–1315. doi: 10.1016/0002-9149(93)90304-u 8256711

[45] Lazzeri C, La Villa G, Barletta G, Franchi F. 24-hour heart rate variability in patients with vasovagal syncope. Pacing Clin Electrophysiol. 2000;23(4 Pt 1):463–468. doi: 10.1111/j.1540-8159.2000.tb00828.x 10793435

[46] Task Force for the Diagnosis and Management of Syncope, European Society of Cardiology (ESC), European Heart Rhythm Association (EHRA), Heart Failure Association (HFA), Heart Rhythm Society (HRS), Moya A, et al. Guidelines for the diagnosis and management of syncope (version 2009). Eur Heart J. 2009;30(21):2631–2671. doi: 10.1093/eurheartj/ehp298 19713422 PMC3295536

[47] Kress AK, Schneider G, Pichler K, Kalmer M, Fleckenstein B, Grassmann R. Elevated cyclic AMP levels in T lymphocytes transformed by human T-cell lymphotropic virus type 1. J Virol. 2010;84(17):8732–8742. doi: 10.1128/JVI.00487-10 20573814 PMC2918996

[48] Small KM, Brown KM, Forbes SL, Liggett SB. Polymorphic deletion of three intracellular acidic residues of the alpha 2B-adrenergic receptor decreases G protein-coupled receptor kinase-mediated phosphorylation and desensitization. J Biol Chem. 2001;276(7):4917–4922. doi: 10.1074/jbc.M008118200 11056163

[49] Calebiro D, Nikolaev VO, Persani L, Lohse MJ. Signaling by internalized G-protein-coupled receptors. Trends Pharmacol Sci. 2010;31(5):221–228. doi: 10.1016/j.tips.2010.02.002 20303186

[50] Hosono M, Takahira T, Fujita A, Fujihara R, Ishizuka O, Tatee T, et al. Cardiovascular and adenylate cyclase stimulant properties of NKH477, a novel water-soluble forskolin derivative. J Cardiovasc Pharmacol. 1992;19(4):625–634. doi: 10.1097/00005344-199204000-00021 1380607

[51] Brignole M, Sutton R, Menozzi C, Garcia-Civera R, Moya A, Wieling W, et al. Early application of an implantable loop recorder allows effective specific therapy in patients with recurrent suspected neurally mediated syncope. Eur Heart J. 2006;27(9):1085–1092. doi: 10.1093/eurheartj/ehi842 16569653

[52] Edvardsson N, Frykman V, van Mechelen R, Mitro P, Mohii-Oskarsson A, Pasquie JL, et al. Use of an implantable loop recorder to increase the diagnostic yield in unexplained syncope: results from the PICTURE registry. Europace. 2011;13(2):262–269. doi: 10.1093/europace/euq418 21097478 PMC3024039

[53] Boersma L, Mont L, Sionis A, García E, Brugada J. Value of the implantable loop recorder for the management of patients with unexplained syncope. Europace. 2004;6(1):70–76. doi: 10.1016/j.eupc.2003.09.006 14697729

[54] Krahn AD, Klein GJ, Yee R, Skanes AC. Randomized assessment of syncope trial: conventional diagnostic testing versus a prolonged monitoring strategy. Circulation. 2001;104(1):46–51. doi: 10.1161/01.cir.104.1.46 11435336

[55] Brignole M, Deharo JC, Menozzi C, Moya A, Sutton R, Tomaino M, et al. The benefit of pacemaker therapy in patients with neurally mediated syncope and documented asystole: a meta-analysis of implantable loop recorder studies. Europace. 2018;20(8):1362–1366. doi: 10.1093/europace/eux321 29267867

[56] Haïssaguerre M, Shoda M, Jaïs P, Nogami A, Shah DC, Kautzner J, et al. Mapping and ablation of idiopathic ventricular fibrillation. Circulation. 2002;106(8):962–967. doi: 10.1161/01.cir.0000027564.55739.b1 12186801

[57] Kapoor WN. Evaluation and management of the patient with syncope. JAMA. 1992;268(18):2553–2560. doi: 10.1001/jama.1992.03490180085031 1404823

[58] Meinkoth JL, Alberts AS, Went W, Fantozzi D, Taylor SS, Hagiwara M, et al. Signal transduction through the cAMP-dependent protein kinase. Mol Cell Biochem. 1993;127–128:179–186. doi: 10.1007/BF01076769 7935349

[59] Gray PC, Scott JD, Catterall WA. Regulation of ion channels by cAMP-dependent protein kinase and A-kinase anchoring proteins. Curr Opin Neurobiol. 1998;8(3):330–334. doi: 10.1016/s0959-4388(98)80057-3 9687361

[60] Willoughby D, Cooper DM. Organization and Ca2+ regulation of adenylyl cyclases in cAMP microdomains. Physiol Rev. 2007;87(3):965–1010. doi: 10.1152/physrev.00049.2006 17615394

[61] Billington CK, Penn RB. Signaling and regulation of G protein-coupled receptors in airway smooth muscle. Respir Res. 2003;4(1):2. 12648290 PMC152647

[62] Brass LF, Laposata M, Banga HS, Rittenhouse SE. Regulation of the phosphoinositide hydrolysis pathway in thrombin-stimulated platelets by a pertussis toxin-sensitive guanine nucleotide-binding protein. Evaluation of its contribution to platelet activation and comparisons with the adenylate cyclase inhibitory protein, Gi. J Biol Chem. 1986;261(36):16838–16847. doi: 10.1016/S0021-9258(19)75964-X 3023367

[63] Masuo K. Roles of beta2- and beta3-adrenoceptor polymorphisms in hypertension and metabolic syndrome. Int J Hypertens. 2010;2010:832821. doi: 10.4061/2010/832821 20981286 PMC2963125

[64] Insel PA, Ostrom RS. Forskolin as a tool for examining adenylyl cyclase expression, regulation, and G protein signaling. Cell Mol Neurobiol. 2003;23(3):305–314. doi: 10.1023/a:1023684503883 12825829 PMC11530207

[65] Torphy TJ. β-Adrenoceptors, cAMP and airway smooth muscle relaxation: challenges to the dogma. Trends Pharmacol Sci. 1994;15(10):370–374. doi: 10.1016/0165-6147(94)90157-0 7809952

[66] Hong F, Brizendine RK, Carter MS, Alcala DB, Brown AE, Chattin AM, et al. Diffusion of myosin light chain kinase on actin: a mechanism to enhance myosin phosphorylation rates in smooth muscle. J Gen Physiol. 2015;146(4):267–280. doi: 10.1085/jgp.201511483 26415568 PMC4586593

[67] Davare MA, Avdonin V, Hall DD, Peden EM, Burette A, Weinberg RJ, et al. A β^2^ adrenergic receptor signaling complex assembled with the Ca^2+^ channel Ca_v_1.2. Science. 2001;293(5527):98–101. doi: 10.1126/science.293.5527.98 11441182

[68] Wang Q, Dai X, Yang W, Wang H, Zhao H, Yang F, et al. Caffeine protects against alcohol-induced liver fibrosis by dampening the cAMP/PKA/CREB pathway in rat hepatic stellate cells. Int Immunopharmacol. 2015;25(2):340–352. doi: 10.1016/j.intimp.2015.02.012 25701503

[69] Huang W, Cane MC, Mukherjee R, Szatmary P, Zhang X, Elliott V, et al. Caffeine protects against experimental acute pancreatitis by inhibition of inositol 1,4,5-trisphosphate receptor-mediated Ca2+ release. Gut. 2017;66(2):301–313. doi: 10.1136/gutjnl-2015-309363 26642860 PMC5284483

[70] Horrigan LA, Kelly JP, Connor TJ. Immunomodulatory effects of caffeine: friend or foe? Pharmacol Ther. 2006;111(3):877–892. doi: 10.1016/j.pharmthera.2006.02.002 16540173

