# Supplementary material for: Diagnosis and prevention of the vasodepressor type of neurally mediated syncope in Japanese patients
Source: PLoS One. 2021 Jun 25;16(6):e0251450. doi: 10.1371/journal.pone.0251450 (PMC8232444; doi:10.1371/journal.pone.0251450)
Supplement: S1 Table — (DOCX) [file pone.0251450.s001.docx]

**S1 Table.** Characteristics of all patients.

|  | All (n=124) | VT (n=45) | Negative (n=33) |
| --- | --- | --- | --- |
| Age (years), mean | 49.3±21.6 | 51.4±23.5 | 54.5±19.0 |
| Male, n (%) | 83 (66.9) | 30 (66.7) | 24 (72.7) |
| Comorbid conditions, n (%) | 95 (76.6) |  |  |
| Hypertension, n (%) | 29 (23.4) | 10 (22.2) | 11(33.3) |
| Diabetes mellitus, n (%) | 17 (13.7) | 4 (8.9) | 7 (21.2) |
| Cardiac disease |  |  |  |
| Ischemic heart disease, n (%) | 10 (8.1) | 5 (11.1) | 3 (9.1) |
| Non ischemic heart disease, n (%) | 9 (7.3) | 3 (6.7) | 3 (9.1) |
| Arrhythmia, n (%) | 28 (22.6) | 10 (22.2) | 9 (27.3) |
| Neurological disease, n (%) | 11 (8.9) | 8 (17.8) | 2 (6.1) |
| Mental disease, n (%) | 15 (12.1) | 5 (11.1) | 4 (12.1) |
| Chronic kidney disease, n (%) | 4 (3.2) | 1 (2.2) | 2 (6.1) |
| Medicine |  |  |  |
| Ca blocker, n (%) | 22 (17.7) | 7 (15.6) | 9 (27.3) |
| β blocker, n (%) | 12 (9.7) | 7 (15.6) | 3 (9.1) |
| ACE/ARB, n (%) | 30 (24.2) | 11 (24.4) | 12 (36.4) |
| Diuretic, n (%) | 4 (3.3) | 1 (2.3) | 3 (9.1) |
| Antiarrhythmic drug, n (%) | 6 (4.9) | 3 (6.7) | 1 (3.0) |
| Antipsychotic/sleeping drug, n (%) | 13 (10.5) | 4(8.9) | 5 (15.2) |
| EF (%), mean | 67.8±8.8 | 69.2±8.9 | 65.3±10.4 |
| (base) |  |  |  |
| Pulse (bpm) | 66.4±14.0 | 66.3±14.4 | 66.5±12.5 |
| Systolic BP (mmHg) | 119.6±17.8 | 118.5±14.1 | 124.3±21.7 |
| Diastolic BP (mmHg) | 73.7±12.6 | 74.4±10.2 | 76.1±15.6 |
