# Supplementary material for: Diagnosis and prevention of the vasodepressor type of neurally mediated syncope in Japanese patients
Source: PLoS One. 2021 Jun 25;16(6):e0251450. doi: 10.1371/journal.pone.0251450 (PMC8232444; doi:10.1371/journal.pone.0251450)
Supplement: S2 Table — (DOCX) [file pone.0251450.s002.docx]

**S2 Table.** Raw data for Holter ECG of 30 VT -NMS patients.

|  | **VT n=19** | **Negative n=11** | ***p* value** |
| --- | --- | --- | --- |
| Age, years | 46.8±21.4 | 54.7±14.5 | 0.289 |
| Male, n (%) | 14(73.7%) | 10(90.9) | 0.047 |
|  |  |  |  |
| Maximum bpm | 120.5±21.7 | 125.2±16.9 | 0.547 |
| Minimum bpm | 72.9±120.0 | 46.7±8.8 | 0.479 |
| Average bpm | 65.5±8.2 | 69.3±8.8 | 0.254 |
| **PVC** | 6.6±10.7 | 611.7±1061.9 | **0.018 *** |
| SVPC | 288.2±1036.1 | 36.8±46.3 | 0.478 |
|  |  |  |  |
| LP, Positive (%) | 12(63.2) | 3(27.3) | 0.089 |
| LP fluctuation,  Positive (%) | 1(5.26) | 3(27.3) | 0.112 |
|  |  |  |  |
| f-QRS min, ms | 106.1±16.7 | 102.2±13.2 | 0.516 |
| f-QRS max, ms | 120.4±19.0 | 113.2±15.1 | 0.292 |
| f-QRS AV, ms | 113.4±18.6 | 107.6±14.0 | 0.378 |
| f-QRS CV, ms | 6.2±26.1 | 0 | 0.443 |
|  |  |  |  |
| LAS40 min, ms | 53.6±81.5 | 21.6±10.2 | 0.209 |
| LAS40 max, ms | 39.3±2.4 | 35.5±3.1 | 0.352 |
| LAS40 AV, ms | 32.7±9.0 | 59.5±105.9 | 0.289 |
| LAS40 CV, ms | 1.7±6.7 | 0.14±0.1 | 0.446 |
|  |  |  |  |
| RAS40 min, μv | 27.2±35.0 | 36.4±36.8 | 0.504 |
| RAS40 max, μv | 53.4±57.5 | 117.1±189.4 | 0.191 |
| RAS40 AV,μv | 39.1±45.8 | 45.6±40.2 | 0.7 |
| RAS40 CV, μv | 2.4±9.4 | 0.2±0.1 | 0.448 |
|  |  |  |  |
| Total score | 2.3±5.1 | 0.6±1.0 | 0.306 |
| Total score max | 3.1±2.2 | 1.7±1.9 | 0.098 |
| AV | 2.1±1.9 | 1.1±1.6 | 0.161 |
| CV | 0.4±0.3 | 0.8±1.0 | 0.078 |
|  |  |  |  |
| HRV Positive, n (%) | 6(54.6) | 10(52.6) | 0.919 |
|  |  |  |  |
| RMSSD 24H, | 174.2±517.5 | 68.4±91.1 | 0.510 |
| RMSSD day, | 67.1±94.9 | 60.8±82.3 | 0.855 |
| RMSSD night, | 71.6±82.5 | 82.9±109.4 | 0.751 |
| RMSSD R.C. | 23.6±50.0 | 40.3±63.1 | 0.429 |
|  |  |  |  |
| PNN50 24H | 18.5±21.0 | 19.4±30.4 | 0.920 |
| PNN50 day | 15.8±20.0 | 17.8±30.6 | 0.833 |
| PNN50 night | 27.8±26.9 | 24.5±30.6 | 0.759 |
| PNN50 R.C. | 116.1±202.5 | 215.6±381.0 | 0.356 |
|  |  |  |  |
| SDANN 24H | 141.9±52.0 | 155.4±60.3 | 0.534 |
| SDANN day | 114.1±43.6 | 117.9±46.1 | 0.823 |
| SDANN night | 65.6±18.7 | 63.8±17.4 | 0.799 |
| SDANN R.C. | -39.9±16.4 | -41.5±17.3 | 0.806 |
|  |  |  |  |
| LF 24H | 1759.3±3013.8 | 2506.7±3426.9 | 0.538 |
| LF day | 1676.7±3023.7 | 1217.9±1681.7 | 0.647 |
| LF night | 1804.3±2896.7 | 2712.5±4304.5 | 0.495 |
| LF R.C. | 35.4±110.8 | 95.3±153.8 | 0.226 |
|  |  |  |  |
| HF 24H | 7288.1±26059.5 | 1998.3±4132.3 | 0.512 |
| HF day | 2179.7±6064.5 | 1597.8±3096.3 | 0.770 |
| HF night | 1765.7±3837.8 | 2911.4±5967.6 | 0.526 |
| HF R.C. | 93.2±119.9 | 131.1±233.0 | 0.559 |
|  |  |  |  |
| LF/HF 24H | 2.8±1.4 | 3.0±2.1 | 0.674 |
| LF/HF day | 3.1±1.7 | 3.3±2.2 | 0.833 |
| LF/HF night | 2.4±1.3 | 2.8±2.1 | 0.554 |
| LF/HF R.C. | -10.8±50.7 | -4.4±32.1 | 0.709 |

R.C.: Rate of change
