# Supplementary material for: Diagnosis and prevention of the vasodepressor type of neurally mediated syncope in Japanese patients
Source: PLoS One. 2021 Jun 25;16(6):e0251450. doi: 10.1371/journal.pone.0251450 (PMC8232444; doi:10.1371/journal.pone.0251450)
Supplement: S3 Table — (DOCX) [file pone.0251450.s003.docx]

**S3 Table.** Analysis of EF differences by age group between VT-NMS-positive and VT-NMS-negative patients.

**Youth**

|  | **All(n=42)** | **VT(n=16)** | **negative(n=6)** | ***p* value** |
| --- | --- | --- | --- | --- |
| Age(years), mean | 23.4±5.0 | 22.7±5.2 | 26.0±6.2 | 0.22 |
| Male, n (%) | 22(52.4) | 9(56.3) | 3(50.0) | 0.79 |
| Female, n (%) | 20(47.6) | 7(43.7) | 3(50.0) |  |
| EF (%), mean | 65.6±7.7 | 67.1±10.4 | 62.3±6.2 | 0.32 |
| (base) |  |  |  |  |
| Pulse (bpm) | 69.0±18.7 | 69.6±17.9 | 75.0±22.8 | 0.56 |
| Systolic BP (mmHg) | 112.6±13.1 | 116.3±11.4 | 118.7±14.3 | 0.68 |
| Diastolic BP (mmHg) | 66.8±8.8 | 68.4±6.5 | 73.2±11.8 | 0.23 |

**Middle aged**

|  | **All(n=39)** | **VT(n=9)** | **negative(n=16)** | **P** |
| --- | --- | --- | --- | --- |
| Age(years), mean | 51.0±8.7 | 54.2±9.4 | 51.0±9.7 | 0.43 |
| Male, n (%) | 27(69.2) | 6(66.7) | 11(68.8) | 0.91 |
| Female, n (%) | 12(30.8) | 3(33.3) | 5(31.2) |  |
| EF (%), mean | 68.2±10.5 | 69.8±8.3 | 65.2±13.7 | 0.37 |
| (base) |  |  |  |  |
| Pulse (bpm) | 65.9±10.3 | 62.2±13.0 | 64.9±8.1 | 0.53 |
| Systolic BP (mmHg) | 120.9±22.0 | 110.0±7.4 | 124.0±28.0 | 0.16 |
| Diastolic BP (mmHg) | 78.0±14.7 | 75.0±7.9 | 79.2±19.3 | 0.54 |

**Senior citizens**

|  | **All(n=43)** | **VT(n=20)** | **negative(n=11)** | **P** |
| --- | --- | --- | --- | --- |
| Age(years), mean | 73.0±5.0 | 73,2±5.2 | 75.0±5.7 | 0.37 |
| Male, n (%) | 34(79.1) | 15(75.0) | 10(90.9) | 0.26 |
| Female, n (%) | 9(20.9) | 5(25.0) | 1(9.1) |  |
| EF (%), mean | 69.6±7.8 | 70.5±8.3 | 67.0±6.0 | 0.23 |
| (base) |  |  |  |  |
| Pulse (bpm) | 64.2±11.1 | 65.5±11.7 | 64.4±9.5 | 0.79 |
| Systolic BP (mmHg) | 125.3±15.3 | 124.1±16.2 | 127.9±14.0 | 0.52 |
| Diastolic BP (mmHg) | 76.6±10.9 | 78.9±11.5 | 73.1±11.3 | 0.19 |
