# Supplementary material for: Diagnosis and prevention of the vasodepressor type of neurally mediated syncope in Japanese patients
Source: PLoS One. 2021 Jun 25;16(6):e0251450. doi: 10.1371/journal.pone.0251450 (PMC8232444; doi:10.1371/journal.pone.0251450)
Supplement: S4 Table — (DOCX) [file pone.0251450.s004.docx]

**S4 Table.** Rate of change of SBP, DBP, and HR in VT-NMS patients and healthy volunteers.

**SBP base 70° 1 min 2 min 3 min 4 min 5 min 6 min 7 min 8 min 9 min 10 min**

**VT** 116.4 122.4 124.0 122.2 123.9 123.9 118.1 117.1 115.8 113.3 113.5 111.8

**(R.C.)** 　 6 7.6 5.8 7.5 7.5 1.7 0.7 -0.6 -3.1 -2.9 -4.6

**Healthy** 107.9 111.1 106.3 108.1 108.8 98.0 107.1 107.1 104.6 106.4 97.5 101.9

**(R.C.)** 　 3.2 -1.6 0.2 0.9 -9.9 -0.8 -0.8 -3.3 -1.5 -10.4 -6

**DBP base 70° 1 min 2 min 3 min 4 min 5 min 6 min 7 min 8 min 9 min 10 min**

**VT** 74 80.6 82.8 80.6 81 78.8 78.6 77.5 77.9 75.6 76.3 76.5

**(R.C.)** 　 6.6 8.8 6.6 7 4.8 4.6 3.5 3.9 1.6 2.3 2.5

**Healthy** 66.2 79.7 71.3 78.5 78.1 65.8 74.5 74.5 71.4 73.3 69.5 72.4

**(R.C.)** 　 13.5 5.1 12.3 11.9 -0.4 8.3 8.3 5.2 7.1 3.3 6.2

**HR base 70° 1 min 2 min 3 min 4 min 5 min 6 min 7 min 8 min 9 min 10 min**

**VT** 66.7 78.5 80.5 81.8 81 81.3 82.1 84.6 83.8 86.1 84.2 82.1

**(R.C.)** 　 11.8 13.8 15.1 14.3 14.6 15.4 17.9 17.1 19.4 17.5 15.4

**Healthy** 65.7 74.3 73.7 74.4 74.7 79.6 77.1 77.1 77.6 75.7 73.5 75.6

**(R.C.)** 　 8.6 8 8.7 9 13.9 11.4 11.4 11.9 10 7.8 9.9

**SBP 11min 12min 13min 14min 15min 16min 17min 18 min 19 min 20 min**

**VT** 112.0 152.9 113.2 111.5 111.3 113.5 112.6 113.8 109.7 112.3

**(R.C.)** -4.4 36.5 -3.2 -4.9 -5.1 -2.9 -3.8 -2.6 -6.7 -4.1

**Healthy** 93.0 102.8 105.3 100.5 104.4 95.3 100.2 101.6 104.8 101.9

**(R.C.)** -14.9 -5.1 -2.6 -7.4 -3.5 -12.6 -7.7 -6.3 -3.1 -6

**DBP 11 min 12 min 13 min 14 min 15 min 16 min 17 min 18 min 19 min 20 min**

**VT** 75.0 75.5 75.8 76.8 74.3 76.6 75.6 76.5 75.3 76.8

**(R.C.)** 1 1.5 1.8 2.8 0.3 2.6 1.6 2.5 1.3 2.8

**Healthy** 59.0 72.5 75.8 71.5 73.6 68.3 72.4 73.7 75.3 74.7

**(R.C.)** -7.2 6.3 9.6 5.3 7.4 2.1 6.2 7.5 9.1 8.5

**HR 11 min 12 min 13 min 14 min 15 min 16 min 17 min 18 min 19 min 20 min**

**VT** 85.2 86.4 87.2 84.8 84.3 85.9 85.5 88.3 85.5 86.9

**(R.C.)** 18.5 19.7 20.5 18.1 17.6 19.2 18.8 21.6 18.8 20.2

**Healthy** 75.0 78.9 80.3 84.5 78.3 84.0 82.6 79.3 82.8 79.7

**(R.C.)** 9.3 13.2 14.6 18.8 12.6 18.3 16.9 13.6 17.1 14

R.C.: rate of change
