# Supplementary material for: Diagnosis and prevention of the vasodepressor type of neurally mediated syncope in Japanese patients
Source: PLoS One. 2021 Jun 25;16(6):e0251450. doi: 10.1371/journal.pone.0251450 (PMC8232444; doi:10.1371/journal.pone.0251450)
Supplement: S5 Table — (DOCX) [file pone.0251450.s005.docx]

|  |  |  |  |  |  |  |  |  |  |  |  |  |  |  |  |  |  |
| --- | --- | --- | --- | --- | --- | --- | --- | --- | --- | --- | --- | --- | --- | --- | --- | --- | --- |
| **S5 Table.** Raw data for adenylate cyclase activities in healthy volunteers and VT-NMS patients during the HUT test. | | | | | | | | | | | | | | | | | |
| **Adrenaline 10uM** | | |  |  |  |  |  |  |  |  |  |  |  |  |  |  |  |
| **Healthy n=15** | **1** | **2** | **3** | **4** | **5** | **6** | **7** | **8** | **9** | **10** | **11** | **12** | **13** | **14** | **15** |  |  |
|  | C36 | C37 | C38 | C040 | C41 | C43 | C46 | C47 | C048 | C050 | C051 | C052 | C053 | C055 | C057 | **Average** | **SD** |
| **Base** | 0.648401 | 0.318276 | 0.261932 | 0.259273 | 0.111025 | 0.379446 | 0.283347 | 0.204512 | 0.371972 | 0.163163 | 0.416767 | 0.201801 | 0.504962 | 0.328588 | 0.327485 | **0.31873** | **0.136734** |
| **70°** | 0.615054 | 0.278172 | 0.188715 | 0.265434 | 0.104945 | 0.232808 | 0.176941 | 0.309194 | 0.295352 | 0.143949 | 0.37588 | 0.157112 | 0.465157 | 0.24738 | 0.161588 | **0.267845** | **0.135052** |
| **10 min** | 0.520062 | 0.337168 | 0.263434 | 0.381105 | 0.163302 | 0.205381 | 0.147584 | 0.273239 | 0.299688 | 0.203486 | 0.31466 | 0.232742 | 0.446966 | 0.261396 | 0.26862 | **0.287922** | **0.101857** |
| **20 min** | 0.571906 | 0.562986 | 0.336593 | 0.277317 | 0.201285 | 0.276292 | 0.171319 | 0.309766 | 0.304976 | 0.222378 | 0.402894 | 0.234516 | 0.370702 | 0.396983 | 0.406615 | **0.336435** | **0.119131** |

| **NMS (VT) n=27** | **1** | **2** | **3** | **4** | **5** | **6** | **7** | **8** | **9** | **10** | **11** | **12** | **13** | **14** | **15** | **16** |
| --- | --- | --- | --- | --- | --- | --- | --- | --- | --- | --- | --- | --- | --- | --- | --- | --- |
|  | S010 | S011 | S017 | S018 | S020 | S022 | S025 | S027 | S028 | S030 | S032 | S033 | S038 | 15S041 | 15S043 | 15S046 |
| **Base** | 0.676036 | 0.696738 | 0.526726 | 0.462099 | 0.619195 | 0.617245 | 0.380325 | 0.333458 | 0.29156 | 0.364902 | 0.688398 | 0.546487 | 0.202504 | 0.319021 | 0.588554 | 0.505285 |
| **70°** | 0.657167 | 0.62745 | 0.460225 | 0.44446 | 0.508129 | 0.585747 | 0.329431 | 0.286579 | 0.318732 | 0.334241 | 0.598428 | 0.44186 | 0.199594 | 0.361061 | 0.599174 | 0.407463 |
| **10 min** | 0.678469 | 0.75921 | 0.621593 | 0.648548 | 0.691409 | 0.645979 | 0.378521 | 0.340734 | 0.419322 | 0.289854 | 0.723816 | 0.469217 | 0.22502 | 0.288876 | 0.733424 | 0.289248 |
| **20 min** | 0.670621 | 0.79486 | 0.66392 | 0.497933 | 0.657591 | 0.535603 | N/A | 0.31338 | 0.847719 | 0.286675 | 0.668766 | N/A | 0.31616 | 0.330366 | 0.655146 | 0.441955 |

|  | **17** | **18** | **19** | **20** | **21** | **22** | **23** | **24** | **25** | **26** | **27** |  |  |
| --- | --- | --- | --- | --- | --- | --- | --- | --- | --- | --- | --- | --- | --- |
|  | 15S047 | 15S049 | 15S051 | 15S054 | 15S056 | 15S057 | S058 | S059 | S061 | S062 | S063 | **Average** | **SD** |
| **Base** | 0.393769 | 0.274236 | 0.413225 | 0.301723 | 0.30929 | 0.553957 | 0.73808 | 0.864333 | 0.582166 | 0.356236 | 0.207341 | **0.474551** | **0.175819** |
| **70°** | 0.477751 | 0.137737 | 0.318269 | 0.274629 | 0.264906 | 0.585668 | 0.842624 | 0.833327 | 0.67998 | 0.439072 | 0.1563802 | **0.450744** | **0.188552** |
| **10 min** | 0.511671 | 0.221869 | 0.426556 | 0.484724 | 0.41221 | 0.574801 | 0.892959 | 0.786934 | N/A | 0.84548 | 0.197681 | **0.521466** | **0.208491** |
| **20 min** | 0.602632 | 0.183293 | 0.345449 | 0.64513 | 0.441775 | 0.657247 | N/A | 0.844848 | N/A | N/A | 0.148141 | **0.524964** | **0.207213** |
| **N/A: not applicable** | |  |  |  |  |  |  |  |  |  |  |  |  |
